# Dataset on structure and mechanical properties of electrospun polyacrylonitrile nanofibrous mesh reinforced by halloysite nanotubes

**DOI:** 10.1016/j.dib.2018.11.039

**Published:** 2018-11-14

**Authors:** K.L. Goh, M. Makaremi, P. Pasbakhsh, R. De Silva, V. Zivkovic

**Affiliations:** a Newcastle University in Singapore, 172A Ang Mo Kio Avenue 8 #05-01, 567739 Singapore; b Newcastle Research & Innovation Institute (NewRIIS), 80 Jurong East Street 21 #05-04, 609607 Singapore; c Monash University, Advanced Platform Technology, Jalan Lagoon Selatan, 47500 Bandar Sunway, Selangor Darul Ehsan, Malaysia; d Sri Lanka Institute of Nanotechnology (SLINTEC), Nanotechnology & Science Park, Mahawatta, Pitipana,1020 Homagama, Sri Lanka; e School of Engineering, Faculty of Science, Agriculture and Engineering, Newcastle University, Newcastle Upon Tyne NE1 7RU UK

## Abstract

The mechanical properties of electrospun polyacrylonitrile (PAN)-based membranes for ultrafiltration, such as oil–water separation and heavy metals from water, are often characterised in the dry state but little is known about the membrane properties in the hydrated state. This dataset comprised mechanical properties and structure-related properties of electrospun PAN-based membranes. The mechanical dataset described the yield strength and strain, stiffness, resilience energy, fracture strength, strain at fracture and fracture toughness of electrospun neat PAN and halloysite nanotube (HNT) reinforced PAN membranes in both hydrated and dry states. The data related to the hydrated state were derived from direct measurements of the mechanical properties of the PAN-based membrane using a novel environmental micromechanical tester. The structure-related dataset comprised electron micrographs and quantitative measurements (fibre diameter and pore diameter) derived from the micrographs. For further interpretation and discussion of the dataset, the reader is referred to the research data article, “Direct measurement of the elasticity and fracture properties of electrospun polyacrylonitrile/halloysite fibrous mesh in water” (Govindasamy et al., 2014).

**Specifications table**

**Table:** 

Subject area	*Engineering, Composite, Material Science Engineering, Mechanical Properties, Filtration*
More specific subject area	*Elasticity properties, Fracture properties, Hydration, Filtration, Desalination, Oil–water separation*
Type of data	*Table, graph, figure*
How data were acquired	*Microscope (FESEM, Hitachi SU8010), Micromechanical tester (in-house developed)*
Data format	*Filtered, Analyzed*
Experimental factors	*Samples (10 mm by 5 mm) were cut from electrospun mesh (100 mm by 100 mm), randomly selected into 4 groups (sample size/group = 5) for mechanical testing*
Experimental features	*Electrospinning was carried out to process the samples. Scanning and Transmission electron microscopy were performed to derive the structural information. Micromechanical (tensile) testing was performed to derive the elasticity and fracture properties.*
Data source location	*Monash University Malaysia*
	*Newcastle University in Singapore/Newcastle Research & Innovation Institute Singapore*
Data accessibility	*Data are available with this article*
Related research article	K. L. Goh, M. Makaremi, P. Pasbakhsh, R. De Silva, and V. Zirkovic, "Direct measurement of the elasticity and fracture properties of electrospun polyacrylonitrile/halloysite fibrous mesh in water," *Polymer Testing,* Under Review [3]

**Value of the data**

• These data are valuable because (1) PAN is used as a material for ultrafiltration; (2) electrospun PAN/HNT fibrous mesh has been recently proposed as a potential membrane material for ultrafiltration, such as oil–water separation and heavy metals from water.
• The data are original in that it describe the mechanical properties of the neat PAN and PAN/HNT fibrous mesh in dry and hydrated states; the data may be useful in planning new and further experiments related to PAN-based membrane, e.g. extending from a single mesh layer to a laminate, and this could require developing an optimum method for the production of PAN/HNT laminates.
• The data could be potentially valuable as it could be used to (1) inform materials engineer about future design of PAN-based membrane, (2) compare with other types of ultrafiltration-related membrane for further insight, as well as to serve as a benchmark for other researchers, and (3) carry out further analysis using new analytical approaches, e.g. in the area of modelling at molecular and mesoscopic levels, which can possibly open up doors for new collaborations.

## 1 Data

The data are focused on the tensile properties of electrospun PAN-based composite mesh in dry and hydrated states.

The tensile properties may be categorized into elasticity and fracture properties. The properties related to elasticity are yield strength, strain at yield strength, stiffness and resilience strain energy density. The fracture properties are the fracture strength, strain at fracture, and strain energy density to fracture. The data of the mechanical properties are tabulated in Table 3 and plotted in Fig. 3.

In addition, the dataset containing the measured fibre diameter and pore diameter, respectively, of the mesh, as well as the calculation for the predictions of fibre structure of the mesh, stiffness and strength of the mesh, and water flux through the mesh, in dry and hydrated states are also included. The data of the structural properties are tabulated in Table 1 (fibre diameter) and Table 2 (pore diameter) and plotted in Fig. 2.

**Table 1:** Data of normalized frequency of fibre diameter.

*D_f_* (μm)	Normalized frequency, PAN fibrous mesh					Normalized frequency, PAN/HNT fibrous mesh				
	Mesh 1	Mesh 2	Mesh 3	Mean	SE	Mesh 1	Mesh 2	Mesh 3	Mean	SE
0.20	0.00	0.00	0.00	0.00	0.00	0.00	0.00	0.00	0.00	0.00
0.25	0.00	0.00	0.00	0.00	0.00	0.00	0.00	0.00	0.00	0.00
0.30	0.02	0.02	0.00	0.02	0.01	0.00	0.00	0.00	0.00	0.00
0.35	0.00	0.02	0.07	0.03	0.02	0.00	0.00	0.00	0.00	0.00
0.40	0.02	0.22	0.07	0.10	0.05	0.00	0.00	0.00	0.00	0.00
0.45	0.10	0.27	0.27	0.21	0.05	0.04	0.03	0.02	0.03	0.00
0.50	0.29	0.24	0.30	0.28	0.01	0.08	0.10	0.17	0.11	0.03
0.55	0.22	0.17	0.23	0.21	0.02	0.32	0.16	0.17	0.22	0.05
0.60	0.24	0.02	0.03	0.10	0.06	0.32	0.29	0.33	0.31	0.01
0.65	0.02	0.02	0.00	0.02	0.01	0.08	0.29	0.29	0.22	0.07
0.70	0.05	0.00	0.00	0.02	0.01	0.08	0.13	0.02	0.08	0.03
0.75	0.02	0.00	0.03	0.02	0.01	0.08	0.00	0.00	0.03	0.03
0.80	0.00	0.00	0.00	0.00	0.00	0.00	0.00	0.00	0.00	0.00

*D_f_* – fibre diameter; SE – standard error of the mean.

**Table 2:** Data of normalized frequency of pore diameter.

*D_p_* (μm)	Normalized frequency, PAN fibrous mesh					Normalized frequency, PAN/HNT fibrous mesh				
	Mesh 1	Mesh 2	Mesh 3	Mean	SE	Mesh 1	Mesh 2	Mesh 3	Mean	SE
0.00	0.00	0.00	0.00	0.00	0.00	0.00	0.00	0.00	0.00	0.00
1.00	0.02	0.51	0.23	0.25	0.14	0.13	0.08	0.14	0.12	0.02
2.00	0.31	0.46	0.40	0.39	0.04	0.29	0.58	0.49	0.45	0.09
3.00	0.26	0.03	0.29	0.19	0.08	0.25	0.25	0.21	0.24	0.01
4.00	0.17	0.00	0.03	0.07	0.05	0.21	0.08	0.07	0.12	0.04
5.00	0.09	0.00	0.06	0.05	0.03	0.04	0.00	0.02	0.02	0.01
6.00	0.05	0.00	0.00	0.02	0.02	0.08	0.00	0.07	0.05	0.03
7.00	0.02	0.00	0.00	0.01	0.01	0.00	0.00	0.00	0.00	0.00
8.00	0.00	0.00	0.00	0.00	0.00	0.00	0.00	0.00	0.00	0.00
9.00	0.00	0.00	0.00	0.00	0.00	0.00	0.00	0.00	0.00	0.00
10.00	0.03	0.00	0.00	0.01	0.01	0.00	0.00	0.00	0.00	0.00
11.00	0.00	0.00	0.00	0.00	0.00	0.00	0.00	0.00	0.00	0.00
12.00	0.02	0.00	0.00	0.01	0.01	0.00	0.00	0.00	0.00	0.00
13.00	0.00	0.00	0.00	0.00	0.00	0.00	0.00	0.00	0.00	0.00
14.00	0.02	0.00	0.00	0.01	0.01	0.00	0.00	0.00	0.00	0.00
15.00	0.02	0.00	0.00	0.01	0.01	0.00	0.00	0.00	0.00	0.00
16.00	0.00	0.00	0.00	0.00	0.00	0.00	0.00	0.00	0.00	0.00

*D_p_* – pore diameter; SE – standard error of the mean.

For further interpretation and discussion of the dataset, the reader is referred to the researchv data article [1].

## 2 Experimental design, materials and methods

### 2.1 Preparation of materials

The structural and mechanical data were derived from the electrospun PAN and PAN/HNT fibrous meshes which we have prepared in our laboratories. A detailed account of the preparation of the solutions of PAN and PAN/HNT used in the electrospinning process have been reported in the research data article [1]. The research data article [1] also contained a detailed account of the process of electrospinning the PAN and PAN/HNT fibrous meshes. Tables 1 and 2, as well Fig. 2, reveal structural data concerning fibre size of nanometer lengthscale and pore size of micrometer lengthscale; in order to achieve these structural features, the following key processing parameters were used in the electrospinning process: solution flow rate = 1.4 mL/h; distance between spinneret and collector = 15 cm; drum rotational speed = 300 rpm; voltage = 13–14 kV.

The data that were collected from the respective structural and mechanical tests involved testing rectangular specimens (10 mm by 5 mm) prepared from each mesh (which featured a size of 100 mm by 100 mm) using a surgical scalpel. The thickness of the specimens, ranging 0.01–0.05 mm, was recorded under a microscope. To account for possible variability in the inherent non-uniformities in the distribution of the fibres throughout a mesh, a randomized procedure was used to identify and allocate specimens from the meshes to each treatment, i.e. dry and hydrated states. For electron microscopy, three specimens from each of the three respective (PAN and PAN/HNT) meshes were used. For mechanical testing in dry condition, five PAN and PAN/HNT specimens were used; similarly, five PAN and PAN/HNT specimens were used for testing in hydrated condition.

### 2.2 Electron microscopy

**Fig. 1:**
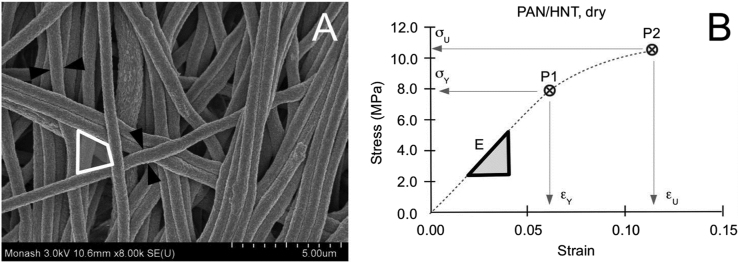
Methodology. (A) Scanning electron micrograph of a PAN/HNT fibrous mesh. The arrows indicate the thickness of the fibre. The polygon encloses the pore area. (B) Plot of stress versus strain derived from a PAN/HNT fibrous mesh in the dry state. The triangle indicates the slope of the linear region which was used to parameterize the stiffness of the mesh (E). P1 and P2 represent the yield point and the fracture point. Symbols *σ_Y_* and *σ_U_* represent the yield strength and fracture strength, respectively; symbols *ε_Y_* and *ε_U_* represent the yield strain and the strain at maximum stress, respectively. The areas from the origin to P1 and P2 correspond to the strain energy density for resilience and fracture toughness, respectively.

The fibre structural data were acquired using ultra-high resolution field emission scanning electron microscope (FESEM, Hitachi SU8010). The images were captured at voltages ranging from 3 kV to 5 kV depending on the magnification (ranging 5.0–10.0 k), from scanning electrons over a thin layer of platinum coated onto the mesh specimens. The STEM mode of the same FESEM instrument was used to record the structure and dispersion of HNTs on or within the fibre. The images were associated with regions identified by a randomised procedure. In this randomised procedure, the field-of-view of each specimen observed under the electron microscope was divided equally into sectors. A randomized procedure was applied to select regions within the field of view for the purpose of recording the images for further analysis. The quantitative structure data, namely fibre thickness and pore size, were derived as shown in Fig. 1A. Open-sourced software, ImageJ was used to carry out the measurements. Individual fibres at different depths were identified and the thickness was measured. The fibre thickness was identified with the fibre diameter (*D_f_*), assuming that each fibre had a circular cross-section. Pores at different depths were identified by the region enclosed by adjacent fibres and outlined to determine the pore perimeter. The pore perimeter was identified with *πD_p_* (each pore was modelled by an equivalent circle) and the pore size was parameterized by *D_p_*. From the value of the pore perimeter, the *D_p_* was found by dividing *πD_p_* by *π*.

**Fig. 2:**
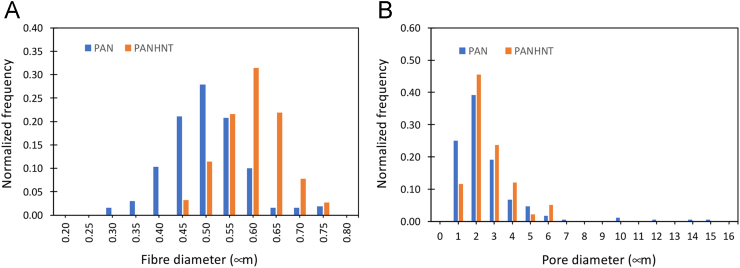
Histograms of the structural properties of PAN and PAN/HNT fibrous meshes. (A) Barchart of normalized frequency versus fibre diameter. (B) Barchart of normalized frequency versus pore diameter.

The data of the structural properties are tabulated in Table 1 (fibre diameter) and Table 2 (pore diameter) and plotted in Fig. 2.

### 2.3 Micromechanical testing

The data on the mechanical properties of the fibrous mesh were acquired by subjecting mesh specimens to tensile loads until rupture using a high-throughput small-scale environmental (horizontal, in-house developed) micromechanical tester. The data comprised two categories of mechanical parameter, namely the elasticity and fracture properties. The data associated with the elasticity-related properties involved the measurements of the yield strength (*σ_Y_*), yield strain (*ε_Y_*), modulus of elasticity (or stiffness; *E*) and strain energy density for resilience (*u_Y_*). The data associated with the fracture properties involved the measurements of the fracture strength (*σ_U_*), fracture strain (*ε_U_*) and fracture toughness (*u_F_*). Fig. 1B shows a sketch of the stress-strain graph to illustrate how the respective mechanical parameters were determined. A straight line was fitted to the points along the initial part of the stress–strain curve where linearity was observed. The gradient of the linear region was used to parameterize the *E*. The *σ_Y_* and *ε_Y_* were identified at the point (i.e. the yield point) immediately after the limit of proportionality. In addition, the area under the curve from the origin to the yield point was determined to parameterize the *u_Y_*. The *u_F_* was determined from the area under the curve from the origin to the point of rupture. The mechanical data described above was derived by testing individual specimens [2], [3]. Individual mesh specimens, gripped at the ends by clamps on the mechanical tester (i.e. the specimen first was mounted-secured using cyanoacrylate adhesive-across a rectangular aluminium frame), were stretched to rupture at a displacement rate of 3.6 mm/min, i.e. 0.06 mm/s following a procedure described elsewhere [3], [4].

The data for the cross-sectional area (*A*) of each specimen, which was used in the calculation for the nominal stress generated in the mesh, involved measuring the thickness and width at the mid-substance (between the grips) of each specimen under an inverted optical microscope, prior to mechanical testing. A setup of the micromechanical tester under the inverted optical microscope has been illustrated in the research article [1]. The data derived from hydrated PAN and PAN/HNT fibrous mesh involved submerging the respective individual specimens in water (held in a pedri dish) during the test.

The stress–strain data that were used to derive the respective mechanical properties was determined from the force (*F*) generated by the specimen versus grip-to-grip displacement (*Δ*). The *F* and *Δ* data for each specimen was recorded by the load cell and the strain transducer of the mechanical tester. Thereafter, the corresponding nominal stress (=*F*/*A*) and strain (=*Δ*/*L_g_*) were determined. Here, *L_g_* is the gauge length of the specimen which corresponds to the initial grip-to-grip distance (adjusted until just before the specimen became taut).

**Fig. 3:**
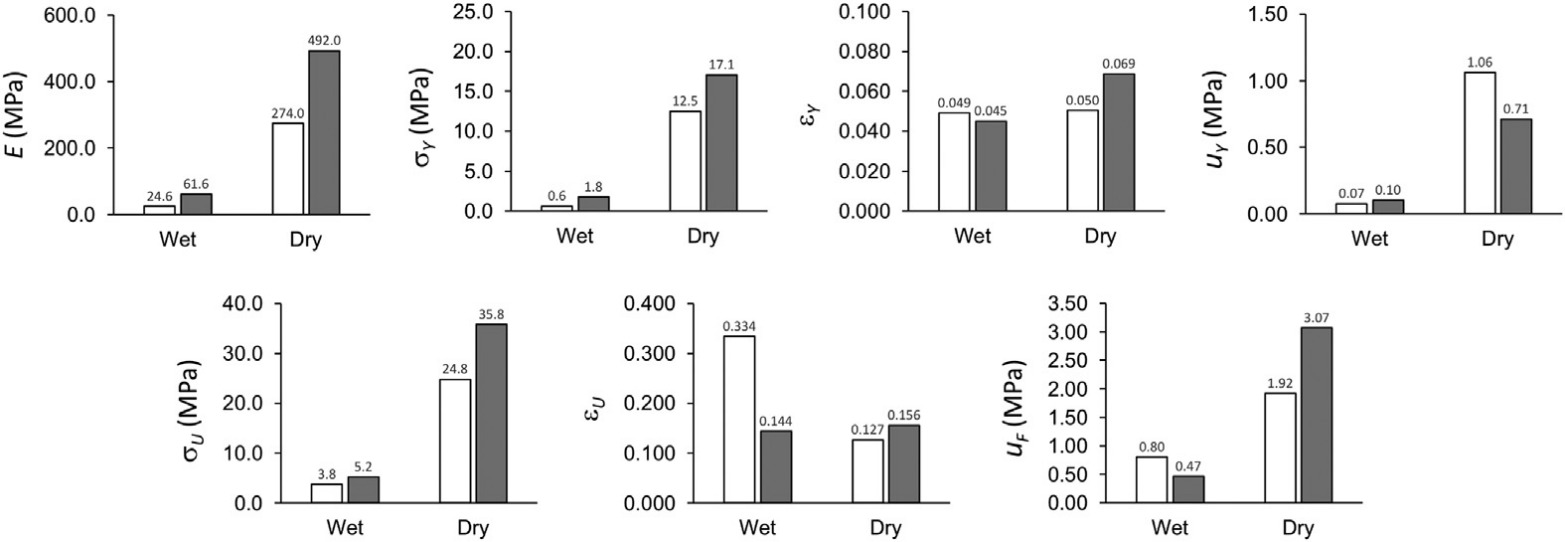
Graphs of the mechanical properties of PAN and PAN/HNT fibrous meshes. Bar with gray shade represents PAN/HNT membrane; unshaded bar represents PAN membrane. Symbol *E* represents the stiffness of the mesh; *σ_Y_* and *σ_U_* represent the yield strength and fracture strength, respectively; *ε_Y_* and *ε_U_* represent the yield strain and the strain at maximum stress, respectively; *u_Y_* and *u_F_* represent the strain energy density for resilience and fracture toughness, respectively.

The data of the mechanical properties are tabulated in Table 3 and plotted in Fig. 3.

**Table 3:** Mechanical properties of PAN and PAN/HNT fibrous meshes in wet and dry states.

	**Wet state**				**Dry state**				**Wet state**			**Dry state**		
	***E***	***σ**_**Y**_*	***ε**_**Y**_*	***u**_**Y**_*	***E***	***σ**_**Y**_*	***ε**_**Y**_*	***u**_**Y**_*	***σ**_**U**_*	***ε**_**U**_*	***u**_**F**_*	***σ**_**U**_*	***ε**_**U**_*	***u**_**F**_*
	**MPa**	**MPa**		**MPa**	**MPa**	**MPa**		**MPa**	**MPa**		**MPa**	**MPa**		**MPa**
PAN	34.6	1.1	0.087	0.03	148.0	1.1	0.011	0.20	3.4	0.235	0.41	8.1	0.142	0.81
	28.3	0.3	0.011	0.06	346.0	39.7	0.115	1.30	3.8	0.306	0.76	58.6	0.169	4.96
	19.5	1.6	0.131	0.02	170.0	1.8	0.011	0.50	3.8	0.366	0.84	10.4	0.082	0.48
	21.8	0.1	0.011	0.08	102.0	6.9	0.060	0.60	3.7	0.431	1.12	9.7	0.109	0.64
	18.9	0.0	0.005	0.18	604.0	13.0	0.055	2.70	4.1	0.333	0.89	37.1	0.131	2.69
														
PANHNT	55.1	1.0	0.022	0.13	767.0	17.3	0.076	1.02	4.0	0.093	0.23	42.1	0.115	1.67
	65.0	2.6	0.066	0.08	661.0	25.0	0.087	0.54	5.2	0.191	0.64	51.6	0.197	5.68
	65.8	2.4	0.055	0.09	746.0	33.9	0.098	0.69	5.5	0.098	0.31	62.3	0.180	5.77
	57.5	2.4	0.066	0.12	129.0	4.7	0.044	0.73	6.1	0.175	0.66	12.5	0.142	1.13
	64.8	0.4	0.016	0.11	157.0	4.4	0.038	0.58	5.4	0.164	0.49	10.6	0.147	1.09

*E* – stiffness, *σ_Y_* – yield strength, *ε_Y_* – yield strain, *u_Y_* – resilience energy, *σ_U_* – fracture strength, *ε_Y_* – strain at fracture strength, *u_F_* – fracture toughness.

### 2.4 Analysis

This section is concerned with the data on the key predictions of the theoretical models used in the research data article [1]. A detailed explanation of how the key predictions were obtained had been presented in the discussion section in the research article [1]. The computation of these predictions can be found in a MS Excel file (analysis_dib.xls). The file contains three worksheets as follows.

The worksheet ׳Fibre Structural Analysis׳ shows a simple computation of the predictions relating to the structural properties of the PAN/HNT meshes, namely the weighted coefficients, *α*_HNT_ and *α*_PAN_, associated with the respective volume fractions of HNT (*V*_HNT_) and PAN (*V*_PAN_), the *V*_HNT_ and *V*_PAN_ in the respective dry and wet states, as well as the *V*_HNT_/*V*_PAN_ in the respective dry and wet states.

The worksheet ׳Stiffness and Strength׳ shows a simple computation of the predictions of the stiffness and fracture strength of the PAN/HNT mesh in the dry and wet states. The Halpin–Tsai model [5] was used in the prediction of the stiffness of the PAN/HNT mesh [6]. The rule-of-mixture model [7] was used in the prediction of the fracture strength of the PAN/HNT mesh [8], [9].

The worksheet ׳Water Flux Analysis׳ shows a simple computation of the predictions of the water flux, *J_w_*, for the PAN and PAN/HNT meshes, as well as the ratio of *J_w_* of PAN/HNT mesh to the PAN mesh, for the respective oil–water separation and desalination cases. A solution diffusion model [10] was used in the prediction of the *J_w_*.

The following paragraphs are concerned with technical explanations about the data reported here.

With regards to data arising from direct measurement of the reinforcing properties-such as the interfacial adhesion properties and the strength of HNT-of HNT in the PAN fibres, this is not possible with our current instrumentation. We anticipate that these properties, namely the interfacial adhesion properties and the strength of HNT, blended in PAN may someday be measured directly by a pull-out technique based on a similar principle that has been used to measuring the interfacial properties of fibre reinforcing composite [8].

With regards to the data on SEM images showing the fractured morphology of the specimens, our observation of the fractured specimens, based on a qualitative examination, revealed that the fracture morphology from SEM images was not appreciably different from that of the morphology of pristine specimens. Thus, we have not included the data with the manuscript because (1) this does not add anything new to the work [1] which preceded this paper and (2) no quantitative assessment was carried out to assess the true extent of the similarities between the morphology of the pristine and the fractured specimen.

With regards to the specimens used for deriving the mechanical data, the specimen dimensions were established on the basis of practicality, i.e. to enable each specimen to be mounted on the micromechanical tester. Thus, the specimen width was smaller than the width of the grips; the specimen length was shorter than the longest distance that the grips could be displaced. We noted that ASTM D1708-13 (Standard Test Method for Tensile Properties of Plastics by Use of Microtensile Specimens) requires that the specimen adopts a dog bone shape; a mould was suggested to produce the dog bone shape specimen. We also noted that D6908-03 (Standard Practice for Integrity Testing of Water Filtration Membrane Systems) did not address micromechanical testing. Of note, producing the dog bone shape specimen requires careful trimming. Otherwise, this could lead to unintended notches-where the narrow width of the specimen joins the boarder width of the specimen-and, consequently, premature failure at the grip end during testing.

With regards to data on contact angle measurement, in this work we have not attempted to assess the hydrophilicity of the membranes by measuring the contact angle, which requires carrying out the wettability test. We noted the following highlights from previous studies: (1) PAN is hydrophilic [11], (3) PAN can be processed to make fibrous membranes—with pore sizes ranging from 0.1 to 0.001 μm—which can be used for ultrafiltration [12], such as water/wastewater treatment and reverse osmosis pretreatment. We also noted that Tripathi and co-workers have assessed the hydrophilicity of PAN membrane as well as PAN composite membrane blended with SiO_2_ nanoparticles [13]. The hydrophilicity of PAN membrane was associated with a contact angle of 68°; PAN/SiO_2_ composite membranes yielded an angle of 32°. Of note, an immersion precipitation method was used to fabricate these membranes [13]. In light of these findings, we anticipated that our PAN/HNT membrane would also exhibit contact angles smaller than PAN membrane since HNT also exhibits hydrophilicity. In the wettability test reported by Tripathi and co-workers [13], they measured the contact angles made by droplets of deionized water (surface tension of 72.8 mN m^−1^) at five different locations on each membrane but they did not mention about recording measurements over time. On this note, as the membrane is porous, it is not entirely clear if it could be a matter of time before the water was absorbed by the membrane. We highlight these issues, namely measurement of contact angle with respect to time and porosity of the membrane, to suggest that the contact angle could change as a function of time. As shown in the research data article [1], the measured porosity did not reveal any appreciable differences in the distribution of pore sizes between the PAN and PAN/HNT fibrous membrane. This suggests that both types of membrane may exhibit similar rate of water absorption. Future experiments could address the measurement of the rate of change of contact angle with time. As the purpose of submitting this data article to Data in Brief is to present the data of an original work reported elsewhere [1], we consider the topic to be beyond the scope of our paper.
